# Energy-Resolved Ultrafast Spectroscopic Investigation on the Spin-Coupled Electronic States in Multiferroic Hexagonal HoMnO_3_

**DOI:** 10.3390/ma15155188

**Published:** 2022-07-26

**Authors:** Wei-Hong Huang, Hao-Keng Wei, Nguyen Nhat Quyen, Pei-Tsung Yang, Yi-Cheng Cheng, Yu-Ting Wang, Ying-Kuan Ko, Chien-Ming Tu, Atsushi Yabushita, Chih-Wei Luo

**Affiliations:** 1 Department of Electrophysics, National Yang Ming Chiao Tung University, Hsinchu 300093, Taiwan; nc7410.sc05@nycu.edu.tw (W.-H.H.); ihatetomato.sc05@nycu.edu.tw (H.-K.W.); nhatquyen126.sc06@nycu.edu.tw (N.N.Q.); crocin201@gmail.com (P.-T.Y.); allen8612231997@gmail.com (Y.-C.C.); wongmom152@yahoo.com.tw (Y.-T.W.); kentko78@gmail.com (Y.-K.K.); yabushita@nycu.edu.tw (A.Y.); 2 Institute of Physics and Center for Emergent Functional Matter Science, National Yang Ming Chiao Tung University, Hsinchu 300093, Taiwan; 3 National Synchrotron Radiation Research Center, Hsinchu 30076, Taiwan; 4 Taiwan Consortium of Emergent Crystalline Materials, Ministry of Science and Technology, Taipei 10601, Taiwan

**Keywords:** multiferroic manganites, antiferromagnetic ordering, ferroelectric ordering, ultrafast spectroscopy

## Abstract

A complete temperature-dependent scheme of the Mn^3+^ on-site d-d transitions in multiferroic hexagonal HoMnO_3_ (*h*-HoMnO_3_) thin films was unveiled by energy-resolved ultrafast spectroscopy. The results unambiguously revealed that the ultrafast responses of the *e*_1g_ and *e*_2g_ states differed significantly in the hexagonal HoMnO_3_. We demonstrated that the short-range antiferromagnetic and ferroelectric orderings are more relevant to the *e*_2g_ state, whereas the long-range antiferromagnetic ordering is intimately coupled to both the *e*_2g_ and *e*_1g_ states. Moreover, the primary thermalization times of the *e*_2g_ and *e*_1g_ states were 0.34 ± 0.08 ps and 0.38 ± 0.08 ps, respectively.

## 1. Introduction

The emergent physical properties resulting from the coupled ferroic orders in multiferroic manganites and their potential applications have attracted considerable research interest [1,2]. In rare-earth manganites, hexagonal *R*MnO_3_ structures with small *R*^3+^ ions (In, Sc, Y, and the lanthanum atoms from Dy to Lu) exhibit coexisting coupled ferroelectric (FE) and antiferromagnetic (AFM) orders [3,4]. In hexagonal HoMnO_3_ (*h*-HoMnO_3_), ferroelectricity occurs below the Curie temperature *T*_C_ (870 K) because structural distortion takes place during the transition from the *P*6_3_/*mmc* to the *P*6_3_*cm* symmetry, as well as the polarization associated with the bonds of Ho and planar oxygen [5]. In *P*6_3_*cm* hexagonal manganites, each ${Mn}^{3+}$ ion is surrounded by five $O^{2-}$ ions, forming triangular planar sub-lattices in the basal plane (*ab*-plane). The magnetic order of ${Mn}^{3+}$ is mainly dominated by antiferromagnetic planar Mn−O−Mn superexchange interactions [6,7]. The AFM spin ordering on the high-spin Mn^3+^ ions occur at the Néel temperature *T*_N_ (76 K). The symmetry of the short-range AFM order of the hexagonal HoMnO_3_ has been derived by second harmonic generation (SHG). Below *T*_N_, the symmetry of the AFM phase is *P*6_3_*cm* and experiences a sudden rotation by an angle of 90° to *P*6_3_*cm* at around 40 K [8].

**Figure 1:**
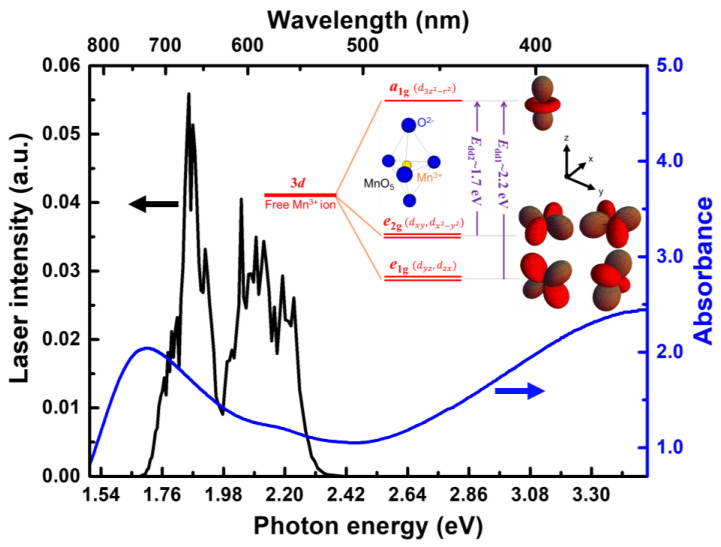
Stationary absorption spectrum of a hexagonal HoMnO_3_ thin film and the laser spectrum used in this study. The inset shows the electronic levels of the five-fold coordinated Mn^3+^ ion in the MnO_5_ trigonal bipyramidal field of the five surrounding O^2−^ ligands.

Hexagonal HoMnO_3_ structures comprise layers of bipyramid MnO_5_ separated by layers of Ho^3+^ ions along the *c*-axis. The Mn^3+^ ions are located near the center of the MnO_5_ bipyramids, forming triangular planar sublattices along the *ab*-plane. Because of the crystal field of the bipyramid structure, the 3*d*-orbit state of the Mn^3+^ ions split into lower-lying doublet states *e*_1g_ ($d_{yz}$/$d_{zx}$) and *e*_2g_ ($d_{xy}$/$d_{x^{2}-y^{2}}$) and an upper-lying singlet state *a*_1g_ ($d_{3z^{2}-r^{2}}$) [9], as shown in the inset of Figure 1. Therefore, the four *d* electrons of Mn^3+^ in the ground state occupy *e*_1g_ and *e*_2g_ and leave *a*_1g_ vacant. Previous studies have determined the band structure of the Mn^3+^ *d* orbits in *R*MnO_3_ (*R* = Gd, Tb, Dy, Ho, Er, and Lu) by using optical absorption spectroscopy [9,10,11,12,13]. The absorption spectrum exhibits two peaks around 1.7 and 2.2 eV corresponding to the transitions from *e*_2g_ to *a*_1g_ and *e*_1g_ to *a*_1g_, respectively, in the on-site Mn^3+^. In addition, the short-range AFM ordering leads to a blue shift in the absorption peaks as the temperature decreases, which further induces a marked change near *T*_N_. The indirect exchange interactions, including double-exchange [14], superexchange [15], and super-superexchange [6], play a key role in explaining the spin-ordering in manganite [16]. Specifically, the magnetic exchange interaction between the Mn^3+^ ions induce the anomalous shift of Mn *d* levels, indicating a strong correlation between the electronic structure and spin ordering [10,12]. Moreover, in addition to hexagonal manganites exhibiting large atomic displacements at *T*_N_ [17,18], the optical phonon frequency also shows an unexpected shift because of the magnetic ordering [19,20,21]. The large atomic displacements combined with phonon anomalies further demonstrate the coupling between the magnetic order and electric dipole moments through the lattice. Accordingly, multiferroic manganites exhibit an intimate coupling between the charge, lattice, and spin degrees of freedom.

Time-resolved optical pump-probe spectroscopy is effective for demonstrating and quantifying the interaction strength among quasiparticles and various degrees of freedom [22,23,24,25,26]. This technique has been extensively employed to identify the underlying physical mechanisms of hexagonal manganites [27,28,29,30,31]. However, most previous studies on transient spectroscopy have focused only on the dynamics of the *e*_2g_ state, and the other unobservable Mn^3+^ *d* orbits remain unclear. In the present study, we adopted an advanced ultrafast spectroscopy technique that involved using a broadband and ultrashort pulse laser to comprehensively examine the complete temperature-dependent scheme of the Mn^3+^ on-site *d-d* transitions in rare-earth multiferroic hexagonal manganite HoMnO_3_.

## 2. Materials and Methods

The samples used in this study were hexagonal *c*-axis HoMnO_3_ thin films with a thickness of 180 nm. The films were deposited on double-sided polished yttria-stabilized zirconia (111) substrates through pulsed-laser (KrF excimer laser) deposition [28]. The thin films were employed to measure both the stationary and transient spectra in a transmissivity configuration to obtain high-quality data. Figure 1 shows the stationary absorption spectrum of the hexagonal HoMnO_3_ thin film measured at room temperature. The absorption spectrum clearly shows the Mn^3+^ *d-d* transition around 1.7 eV (*e*_2g_ to *a*_1g_, *E*_dd2_) and 2.2 eV (*e*_1g_ to *a*_1g_, *E*_dd1_). The transition peak centered at about 1.7 eV is consistent with previous optical absorption spectra in hexagonal-phase *R*MnO_3_ (*R* = Gd, Tb, Dy, Ho, Er, and Lu) [9,10,11,12,13]. The other hidden *d-d* transition around 2.2 eV, which is embedded in the substantially more intense absorption peak, was verified using second-harmonic generation [8,13,32]. To simultaneously reveal the strongly AFM- and temperature-dependent Mn^3+^ *d-d* transitions (i.e., *E*_dd1_ and *E*_dd2_), a light source with a broad spectrum in the visible range is required [33]. The time-resolved spectroscopic measurements in this study were based on 10 fs visible pulses generated by a noncollinear optical parametric amplifier (NOPA) [34,35]. A generative amplifier (800 nm, 5 kHz, 1.8 W, Legend-USP-HE; Coherent, Santa Clara, CA, USA) seeded with a Ti:sapphire laser oscillator (Micra 10; Coherent) was used as the pump source of the NOPA. Figure 1 shows that the laser spectrum (1.7–2.3 eV) covered the targeted whole Mn^3+^ *d-d* transition bands. For the pump-probe measurements, a beam splitter splits the visible pulses into pump and probe beams with the same spectrum. The fluences of pump and probe were 0.85 and 0.07 mJ/cm^2^, respectively, and focused on the samples. The normalized transient transmittance changes Δ*T*/*T* (Δ*T*: the transmittance changes induced by the pump pulses; *T*: the transmittance of the probe pulses) spectra were captured using a wavelength-resolved multichannel lock-in amplifier as a function of delay time between pump and probe pulses [36].

## 3. Results and Discussion

**Figure 2:**
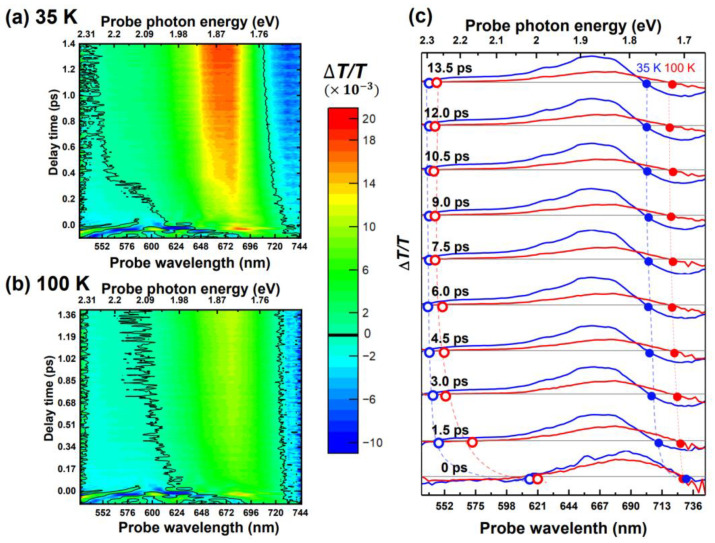
(**a**,**b**) Two-dimensional plots of the transient difference transmittance Δ*T*/*T* at temperatures below (35 K) and above (100 K) *T*_N_. (**c**) Time-resolved Δ*T*/*T* spectra at different delay time between the pump and probe pulses at 35 K (blue) and 100 K (red). The horizontal gray lines show where Δ*T*/*T* = 0. The solid and hollow dots represent the boundary of *d*-*d* transitions, and the solid and hollow dots respectively indicate the time-resolved *E*_dd2_ (*e*_2g_ → *a*_1g_) and *E*_dd1_ (*e*_1g_ → *a*_1g_) transitions. The dashed lines are guides for eyes to represent the time evolution of these transitions.

**Figure 3:**
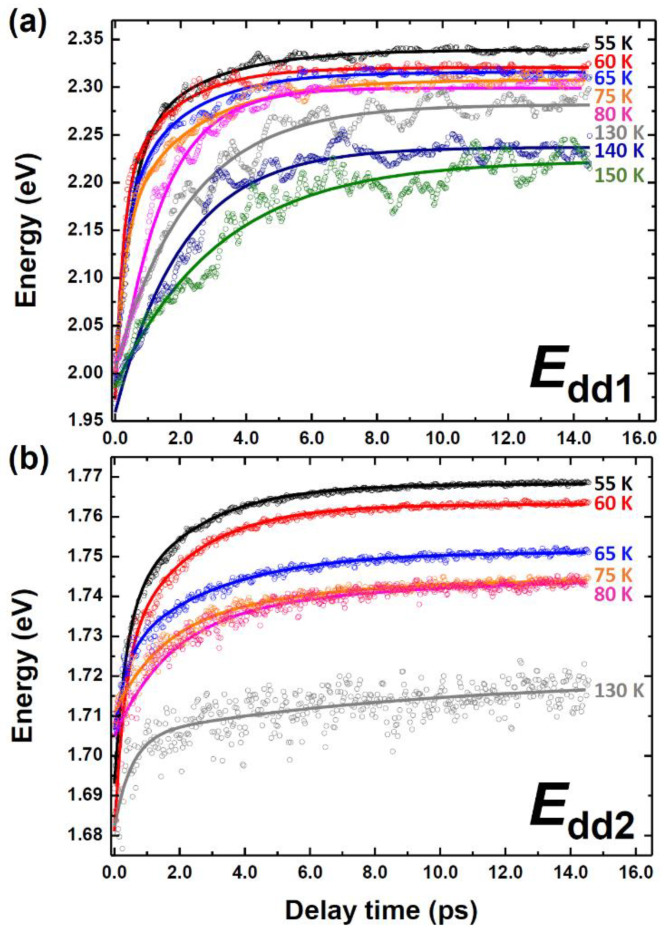
Time evolution of Mn^3+^ on-site *d-d* transition of (**a**) *E*_dd1_ (*e*_1g_ → *a*_1g_) and (**b**) *E*_dd2_ (*e*_2g_ → *a*_1g_) at different temperatures.

Figure 2a,b display the two-dimensional (2D) plots of the relative transient transmittance change (Δ*T*/*T*) spectra as functions of the probe photon energy and delay time at temperatures above (*T* = 100 K) and below (*T* = 35 K) *T*_N_. In the 2D plots, the black lines represent the borders of the positive and negative components of the Δ*T*/*T*(*υ*, *t*) signals. The temperature dependence of the positive Δ*T*/*T* signal in the range of approximately 1.7–2.3 eV was attributed to photobleaching resulting from the depletion of the initial state and the population of the excited state, indicating the *d-d* transitions of *E*_dd1_ and *E*_dd2_. As a result, the energy dependence of the positive Δ*T*/*T* signals (in Figure 2c) is similar to that of the stationary absorption spectrum shown in Figure 1. By contrast, the induced absorption to the higher excited states resulted in a negative Δ*T*/*T* signal in the blocked-photon energy range, which did not correspond to the on-site Mn^3+^ *d-d* transition bands. Therefore, the zero-amplitude position distinctly indicated the boundary of the *d-d* transitions *E*_dd1_ and *E*_dd2_ as the solid black lines in Figure 2a,b. The transition band edges *E*_dd1_ and *E*_dd2_ were extracted to further investigate the transient dynamics of the Mn^3+^ *d* bands at various temperatures, as shown in Figure 3, and both transition bands *E*_dd1_ and *E*_dd2_ clearly exhibited blue shift when the temperature decreased. Furthermore, in addition to the monotonic blue shift, the transient curves revealed the significant characteristics within the short period at temperatures below *T*_N_.

**Figure 4:**
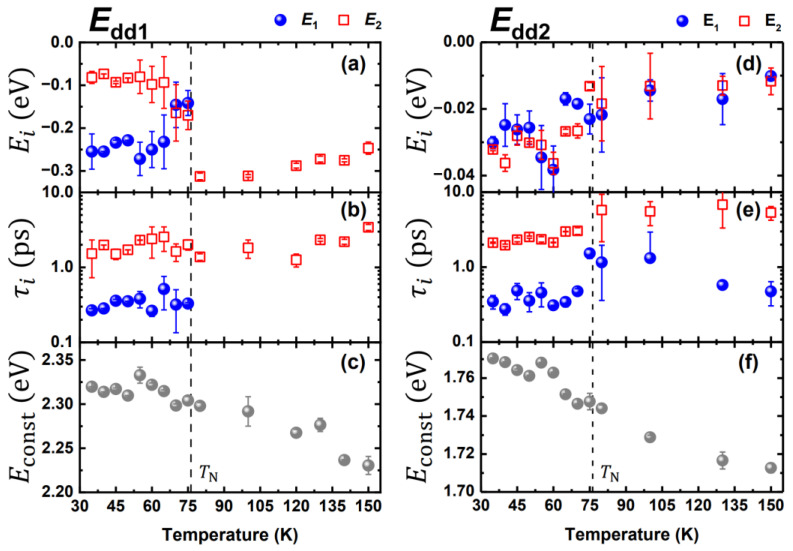
Fitting results of the *E*_dd1_ (*e*_1g_ → *a*_1g_) and *E*_dd2_ (*e*_2g_ → *a*_1g_) spectra in Figure 3 obtained by using Equation (1). (**a**,**d**) Amplitudes *E*_1_ and *E*_2_, (**b**,**e**) relaxation times $\tau_{1}$ and $\tau_{2}$ of *E*_dd1_ and *E*_dd2_ spectra at various temperatures. (**c**,**f**) The constant term of Equation (1) for the energy relaxations in *E*_dd1_ and *E*_dd2_ spectra. The black dashed lines indicate *T*_N_.

The time-resolved traces of *E*_dd1_(*t*) and *E*_dd2_(*t*) at each photon energy level can be phenomenologically expressed as

(1) $$E\left(t\right)=E_{1}e^{-\frac{t}{\tau_{1}}}+E_{2}e^{-\frac{t}{\tau_{2}}}+E_{const},$$

where *E_i_* is the amplitude of the exponential function, and *τ_i_* represents the relaxation time for the corresponding component. Figure 4 shows the fitting results (for the detailed fitting results, please see Table S1 in Supplementary Materials). The constant term *E*_const_ in Figure 4c,f indicates the transition energy level after thermal equilibrium was reached. In consistence with the temperature-dependent stationary absorption spectra in previous studies [10,12], the transition energies shifted and exhibited an anomaly at *T*_N_. In *E*_dd2_ (see Figure 4d,e), both the amplitudes (*E*_1_ and *E*_2_) and time constants (*τ*_1_ = 0.38 ± 0.08 ps and 0.95 ± 0.50 ps; *τ*_2_ = 2.40 ± 0.40 ps and 5.90 ± 0.70 ps; below and above *T*_N_, respectively) exhibited noticeable changes across *T*_N_. On the other hand, the time-dependent *E*_dd1_ (see Figure 4a,b) differed markedly at temperatures above and below *T*_N_. The fast component $\tau_{1}$ (0.34 ± 0.08 fs) was observed only at temperatures below *T*_N_, whereas the slow component $\tau_{2}$ (2.00 ± 0.60 ps) was preserved at all of the measured temperatures.

The assignment of the relaxation components in multiferroic materials has been a challenging subject for decades because of the complicated correlations among the electron, lattice, charge polarization, and AFM spin ordering. A previous study [37] attributed a relaxation time of approximately 0.4 ps to phonon thermalization. On the same time scale, Satoh et al. [38] assigned a relaxation time of approximately 0.9 ps to the demagnetization of AFM compounds. Additionally, previous studies have attributed the few-ps component to electron-lattice relaxation [39,40] or spin-lattice relaxation [28]. In this paper, we propose a model based on our results as well as those from previous studies. The few-ps component $\tau_{2}$ occurred in both *E*_dd1_ and *E*_dd2_ at all of the measured temperatures. Thus, the few-ps component $\tau_{2}$ could be attributed to the relaxation of the excited carriers in *a*_1g_, which is the final state of both *E*_dd1_ and *E*_dd2_ transitions from the initial states $e_{1g}$ and $e_{2g}$, respectively. The excited electrons relaxed to the bottom of *a*_1g_ and banded through the electron–phonon coupling with a few-ps relaxation time, and the transition band exhibited a blue shift induced by the disappearance of the renormalization of the bandgap [41]. This has also been observed in other manganites [27,42]. The significant changes in the amplitudes and relaxation time across *T*_N_ indicate an intimate correlation among the electron, lattice, and spin, which corresponds to the sudden shift in the positions of relevant atoms [17,18] and the anomaly in the Raman spectra [19,20] at the spin-ordering temperature.

In contrast, the sub-ps component $\tau_{1}$ cannot be assigned to population relaxation in the common final state because it exists only in *E*_dd2_ at high temperatures, as shown in Figure 4b,e. Therefore, the sub-ps component in *E*_dd1_ and *E*_dd2_ are ascribed to the relaxation in *e*_1g_ and *e*_2g_, respectively. The *e*_2g_ state comprises the $d_{xy}$ and $d_{x^{2}-y^{2}}$ orbits, which lie on the basal plane. These orbits are strongly hybridized with the planar oxygen of the bipyramid structure, indicating a close correlation with the charge-ordering characteristic of the geometric ferroelectricity. Below the FE transition temperature (*T*_C_ = 870 K in HoMnO_3_), the FE moment is along the *c*-axis between the rare-earth ion (Ho^3+^ in this case) and the planar oxygen on the distorted trigonal bipyramid MnO_5_ [5]. Accordingly, the sub-ps lifetime $\tau_{1}$ is considered to correlate with the destruction of the FE state. Besides, the superexchange in the planar Mn-O-Mn chain combined with the magnetoelastic coupling [17,18] modifies the *e*_2g_ state and induces a significant difference in both the amplitude and lifetime (including $\tau_{1}$ and $\tau_{2}$ in Figure 4d,e) of the pump-probe spectra across *T*_N_, particularly for the sub-ps component $\tau_{1}$ exhibiting the magnetoelectric coupling. Moreover, this sub-ps (0.38 ± 0.08 ps and 0.95 ± 0.50 ps below and above *T*_N_, respectively) component, which is associated with spin ordering, can be observed at temperatures far above *T*_N_, indicating that the *e*_2g_ state essentially couples with the short-range AFM spin ordering, which cannot be reliably obtained from standard magnetization measurements. This is in consistence with the previous results of stationary absorption spectra [9,10,11,12] and our time-resolve spectroscopies [29,31], which have demonstrated that the *e*_2g_ state is highly sensitive to short- and long-range AFM spin ordering.

However, the sub-ps component $\tau_{1}$ can be observed only in the presence of long-range spin ordering in the *e*_1g_ state (Figure 4b). The *e*_1g_ state comprises $d_{yz}$ and $d_{zx}$, which are not as sensitive to the planar oxygen as the *e*_2g_ state. The time-dependent *E*_dd1_ shows significant larger fluctuations at temperatures above *T*_N_ in Figure 3a. According to a previous study [43] on PL, the electronic transfer from *a*_1g_ to *e*_1g_ was strongly blocked by spin fluctuations at temperatures above *T*_N_, indicating that the *E*_dd1_ is dominated by long-range AFM spin ordering. Therefore, the spin-*e*_1g_ orbit interaction was attributed as the main contributor to the sub-ps component in the *e*_1g_ state [44]. Furthermore, the temperature dependence of the Raman-active phonons, inducing anharmonicity in the A_1_ phonon mode (which is the oxygen vibrate along the *c*-axis) below *T*_N_, indicates that the spin-orbit interaction is strongly influenced by the anisotropic superexchange between the Mn^3+^ and Ho^3+^ ions and the super-superexchange of Mn-O-O-Mn along the *c*-axis [19,45,46]. This component $\tau_{1}$ (0.34 ± 0.08 ps) can be ascribed to the thermalization of the spin subsystem in the *e*_1g_ state.

## 4. Conclusions

In summary, we have demonstrated that the Mn^3+^ *d* orbit electronic states are strongly affected by the electric–magnetic coupling in multiferroic *h*-HoMnO_3_ thin films. The 2D energy- and time-resolved spectroscopy measurements carried out at various temperatures have unambiguously disclosed the characteristics of Mn^3+^ *d* orbits. Short-range AFM spin ordering and FE ordering are related to the *e*_2g_ state. By contrast, long-range AFM spin ordering is strongly coupled to both the *e*_2g_ and *e*_1g_ states. The slow electron–phonon relaxation time in the *a*_1g_ state is 2.70 ± 1.50 ps. Moreover, the depolarization time in the *e*_2g_ state above *T*_N_ is 0.95 ± 0.50 ps, and an anomaly is observed at the AFM spin-ordering temperature, further shortening of the fast relaxation time to 0.38 ± 0.08 ps. In addition, the fast spin-thermalization time caused by the spin-orbit (*d*_yz_ and *d*_zx_ orbits) interaction in the *e*_1g_ state is 0.34 ± 0.08 ps. Therefore, this study has demonstrated that magnetic ordering in HoMnO_3_ intimately coupled with the electronic structure of both the *e*_1g_ and *e*_2g_ states, respectively, can be investigated using the proposed energy-resolved ultrafast spectroscopy technique.

## Data Availability

Not applicable.

## Supplementary Materials

The following supporting information can be downloaded at: https://www.mdpi.com/article/10.3390/ma15155188/s1, Table S1: Fitting results of the *E*_dd2_ and *E*_dd1_ spectra in Figure 3 obtained by using Equation (1).
