# Supplementary material for: Energy-Resolved Ultrafast Spectroscopic Investigation on the Spin-Coupled Electronic States in Multiferroic Hexagonal HoMnO3
Source: Materials (Basel). 2022 Jul 26;15(15):5188. doi: 10.3390/ma15155188 (PMC9332413; doi:10.3390/ma15155188)
Supplement: Supplementary file 1 [file materials-15-05188-s001.zip › materials-1801074-supplementary.pdf]

Supplementary Materials:

Table S1. Fitting results of the E_{dd2} and E_{dd1} spectra in Figure 3 obtained by using Eq. (1).

E_{dd1} ($e_{1g} \rightarrow a_{1g}$)

Temp. (K)	E_1 (eV)	E_2 (eV)	E_{const} (eV)	τ_1 (fs)	τ_2 (ps)
35	-0.2547 ± 4.1E-02	-0.0819 ± 1.4E-02	2.3198 ± 7.8E-05	266.80 ± 1.4E+01	1.52 ± 8.0E-01
40	-0.2547 ± 1.3E-05	-0.0736 ± 2.0E-05	2.3139 ± 9.5E-07	283.10 ± 4.4E-02	1.98 ± 5.2E-04
45	-0.2340 ± 5.9E-03	-0.0932 ± 2.9E-03	2.3171 ± 1.3E-03	358.85 ± 1.5E+01	1.51 ± 2.4E-01
50	-0.2285 ± 2.1E-05	-0.0831 ± 2.2E-05	2.3097 ± 5.5E-07	353.18 ± 3.9E-02	1.72 ± 3.5E-04
55	-0.2719 ± 3.9E-02	-0.0802 ± 3.9E-02	2.3328 ± 9.1E-03	381.88 ± 9.4E+01	2.31 ± 1.0E-02
60	-0.2500 ± 4.2E-02	-0.0980 ± 4.2E-02	2.3219 ± 2.0E-03	263.22 ± 4.2E+01	2.40 ± 1.1E+00
65	-0.2320 ± 6.3E-02	-0.0936 ± 6.0E-02	2.3148 ± 1.3E-03	514.70 ± 2.4E+02	2.55 ± 9.2E-01
70	-0.1457 ± 5.3E-02	-0.1645 ± 6.6E-02	2.2984 ± 1.8E-03	319.07 ± 1.8E+02	1.63 ± 4.3E-01
75	-0.1415 ± 2.9E-02	-0.1703 ± 3.3E-02	2.3041 ± 4.8E-03	328.97 ± 1.1E+01	2.01 ± 3.1E-01
80	-	-0.3128 ± 7.7E-03	2.2980 ± 3.9E-03	-	1.38 ± 1.4E-01
100	-	-0.3116 ± 3.2E-03	2.2918 ± 1.7E-02	-	1.82 ± 5.0E-01
120	-	-0.2878 ± 5.6E-03	2.2675 ± 3.9E-03	-	1.26 ± 2.5E-01
130	-	-0.2723 ± 6.8E-03	2.2764 ± 7.5E-03	-	2.33 ± 9.8E-02
140	-	-0.2753 ± 3.6E-03	2.2365 ± 7.7E-04	-	2.20 ± 1.6E-01
150	-	-0.2468 ± 1.4E-02	2.2305 ± 1.0E-02	-	3.42 ± 3.2E-01

E_{dd2} ($e_{2g} \rightarrow a_{1g}$)

Temp. (K)	E_1 (eV)	E_2 (eV)	E_{const} (eV)	τ_1 (fs)	τ_2 (ps)
35	-0.0301 ± 1.8E-03	-0.0322 ± 3.6E-04	1.7704 ± 3.5E-04	346.520 ± 7.0E+01	2.12 ± 3.5E-02
40	-0.0248 ± 6.4E-03	-0.0363 ± 2.5E-03	1.7684 ± 2.8E-04	275.392 ± 4.7E+01	1.95 ± 7.3E-02
45	-0.0262 ± 4.4E-03	-0.0281 ± 2.8E-03	1.7642 ± 6.4E-04	485.113 ± 1.2E+02	2.32 ± 1.4E-01
50	-0.0257 ± 5.1E-03	-0.0302 ± 3.6E-04	1.7612 ± 3.9E-04	354.641 ± 1.0E+02	2.52 ± 8.9E-02
55	-0.0346 ± 9.6E-03	-0.0308 ± 4.4E-03	1.7681 ± 2.1E-04	455.891 ± 1.6E+02	2.35 ± 1.9E-01
60	-0.0383 ± 7.2E-03	-0.0364 ± 3.3E-03	1.7629 ± 4.0E-04	308.325 ± 5.3E-01	2.13 ± 1.7E-02
65	-0.0170 ± 1.9E-03	-0.0268 ± 6.9E-04	1.7515 ± 4.1E-04	342.099 ± 4.7E+01	2.98 ± 1.6E-02
70	-0.0185 ± 1.3E-03	-0.0266 ± 2.1E-03	1.7465 ± 7.9E-05	475.203 ± 3.9E+01	3.05 ± 3.2E-01
75	-0.0231 ± 4.5E-03	-0.0132 ± 1.1E-03	1.7477 ± 4.3E-03	1519.961 ± 2.2E+02	16.16 ± 1.6E+01
80	-0.0218 ± 1.1E-02	-0.0184 ± 1.1E-02	1.7441 ± 1.8E-03	1156.294 ± 8.0E+02	5.77 ± 3.6E+00
100	-0.0145 ± 3.2E-03	-0.0132 ± 9.9E-03	1.7288 ± 1.0E-03	1315.512 ± 1.6E+03	5.52 ± 1.9E+00
130	-0.0171 ± 7.7E-03	-0.0130 ± 2.8E-03	1.7166 ± 4.5E-03	576.011 ± 7.3E+01	6.82 ± 3.5E+00
150	-0.0101 ± 2.1E-05	-0.0117 ± 4.0E-03	1.7127 ± 2.5E-03	471.907 ± 1.7E+02	5.33 ± 1.1E+00
